# On-the-job vocational training of nonprofessional ethnic health workers of a primary health care team improves their sense of coherence

**DOI:** 10.1186/s12960-021-00690-0

**Published:** 2022-02-07

**Authors:** Cintia Katona, Éva Bíró, Szilvia Vincze, Karolina Kósa

**Affiliations:** 1 Department of Behavioural Sciences, Faculty of Medicine, University of Debrecen, Debrecen, Hungary; 2 Department of Preventive Medicine, Faculty of Medicine, University of Debrecen, Debrecen, Hungary; 3 Department of Sectoral Economics and Methodology, Faculty of Economics and Business, University of Debrecen, Debrecen, Hungary

**Keywords:** Roma population, Primary health care, Monitoring, Health status

## Abstract

**Objectives:**

A Primary Care Model Programme had been implemented in Hungary between 2013 and 2017 in which group practices were established that employed—among others—nonprofessional health workers (health mediators, similar to community health workers) to facilitate access for the most disadvantaged population groups. The health of mediators, themselves mostly disadvantaged ethnic Roma, was monitored every odd year of the Programme.

**Methods:**

A repeated cross-sectional health interview survey had been implemented inviting all health mediators who were employed at the time of the survey. The same questionnaire was used in all 3 surveys with items from the European Health Interview Survey 2009 and validated versions of other scales.

**Results:**

Positive changes occurred in the health status of mediators during 5 years of follow-up. Significant improvement in mental health occurred among those who completed on-the-job vocational training. By 2017, significant increase in sense of coherence was observed among those who obtained vocational qualification as opposed to those who did not. The proportion of highly stressed mediators showed a significant increase among those with no vocational training. Improvement was detected in all mediators in health awareness, dysfunctional attitudes, psychological stress and smoking prevalence.

**Conclusions:**

Significant improvement in mental status among those who obtained on-the-job vocational qualification were observed during follow-up of ethnic Roma health mediators in the programme in which they were equal members of the primary health care team. Employment of health mediators in primary care teams not only contributed to improving access to care for disadvantaged groups, but also improved the mental health of mediators themselves.

## Introduction

Many international reports and publications detailed the manifold disadvantages experienced by the Roma, the largest ethnic group in Europe [1–3], and in Hungary [4, 5], that led in 2005 to the launching of the international initiative “Decade of Roma Inclusion” [6]] and in 2011 to the development of national Roma integration strategies in EU member states [7] including Hungary [8]. 

Reduction of economic and social disparities within Hungary, including the Roma, was also one of the main objectives of the Swiss–Hungarian bilateral framework agreement concluded in 2007 [9] that specified four priority areas for funding, human resource and social development as one of them. A large-scale community-oriented programme in primary health care (titled “Primary Care Development Model Programme”) had been implemented in this priority area between 2012 and 2017 in Hungary that gives the framework for the present paper. 

The Model Programme was designed to introduce group practices in primary care to create a new operational model in Hungary (where all general practices were single-handed until then) which would enable general practitioners to employ nonmedical professionals in order to widen primary care services and improve access to and use of these services for all population groups, including disadvantaged, among them Roma, people. Summary [10] and details [11–13], of the Model Programme have been published elsewhere.

Briefly, four group practices (called GP clusters, each formed by six general practitioners) were established in two economically disadvantaged regions of the country. GP clusters funded the employment of nonmedical health professionals such as public health specialists, dietitian, physiotherapist, health psychologist, and as a novelty, GP clusters also employed nonprofessional workers called health mediators specifically for the purpose of facilitating communication and access to primary services for the most disadvantaged population groups. 

Health mediators, similarly to community health workers in the healthcare systems of developing countries [14], had worked in various arrangements in primary care in European countries but for a different reason compared to CHWs. The major task of mediators is to facilitate access of Roma minority groups to healthcare [15] rather than substitute for professional workers. The employment of mediators in the Hungarian Model Programme was supported by positive international experiences [16] including the ROMED initiative of the Council of Europe that was established to train mediators for various settings (healthcare, schools, local authorities, etc.) in order to facilitate communication and cooperation between the Roma and public institutions [17]. 

Employment of 48 health mediators (12 per GP cluster) was planned. Mediators (called “segéd-egészségőrök” in Hungarian) were recruited from the local communities with no requirement for professional or vocational training and were employed half-time (20 h per week) by the GP clusters. Preference was given to those applicants who self-identified as Roma or had experience working with Roma population groups in the serviced areas. They were supervised by the so-called public health coordinator who supervised all nonmedical workers of the team [18]. It is worth noting that the Hungarian Model Programme was Europe-wide the first in which health mediators were employed as equal members of the primary care team, being funded from the same budget and covered by the same obligations and rules as all other members of the GP cluster. 

In order to prepare health mediators for their jobs, mandatory 30-h training in health mediation was provided in 2013 (within one month of the start of employment) and in 2015 by the Hungarian partner organization of the ROMED initiative [19]. To increase their capacity and long-term employment prospects, vocational training in assistant nursing and assistant social services was offered in the first year of employment to all those mediators who did not have any health-related qualification, had the educational requirements to start this training, and volunteered to do so. Altogether 20 persons in assistant nursing and 2 persons in assistant social services obtained this qualification by 2014. Mandatory 1-day trainings were provided to all mediators approximately every half year throughout the Model Programme.

All trainings, including vocational training, were delivered during work hours, took place in Debrecen, geographically away from the workplaces and co-workers of health mediators, and participation was completely free of charge for them. Transfer, accommodation, meals, and all training tools and equipment were covered from the budget of the Model Programme.

Health mediators, being a group of rural and mostly Roma women with low education whose life substantially changed by employment in the Model Programme, presented unique opportunity to monitor what impact employment, higher income, work responsibilities and being part of a professional team would have on their health. Therefore, in accordance with international recommendations [20], the major aims of the Swiss Contribution, and the Hungarian National Social Integration Strategy, and also contributing to the evaluation of the Programme, a project to monitor the health status of mediators throughout the Model Programme was set up. The aim of this paper is to describe the results of this monitoring project. One of the aims was to examine the potential changes in health outcomes of the health mediators over 5 years of their employment; another aim was to investigate the impact of vocational training on their health status.

## Methods

### Study design and participants

**Fig. 1:**
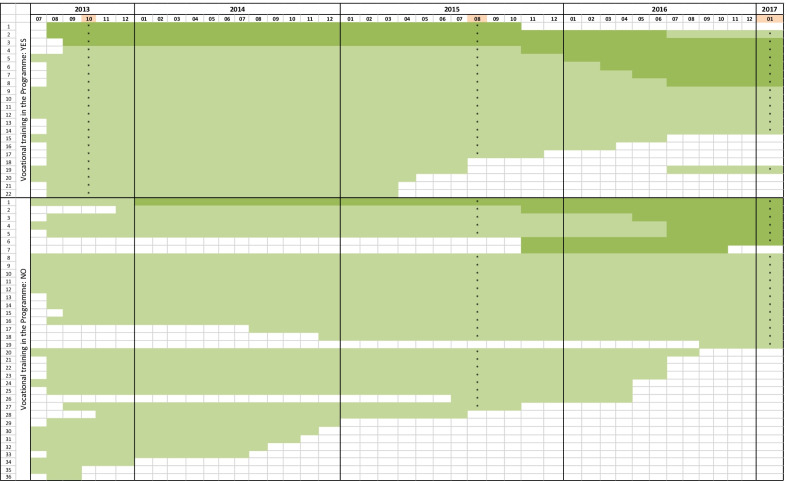
The number of health mediators employed during the Model Programme (light green: half-time, dark green: full time position); mediators invited for health surveys (starred); and the timing of health surveys (months in orange)

The questionnaire-based study had a repeated cross-sectional design. The Programme funded the employment of altogether 48 health mediators, but this number fluctuated during the Programme (Fig. 1). All health mediators employed at the given time of data collection were invited to participate voluntarily and anonymously (since individual identification was not granted by the ethical permission) except in 2013 as shown in Fig. 1. Turnover among the mediators during 5 years of the Programme and the obligation of anonymity excluded panel design.

### Data collection

Data collection was carried out first in October 2013 (1st), repeated in August 2015 (2nd) and January 2017 (3rd) during trainings organized by the University of Debrecen. The first data collection occurred during the vocational training for assistant nursing described above, and those 20 mediators were invited who entered this particular training. The second and third data collection was completed during 1-day trainings that were mandatory for all health mediators. Vocational training had already been completed at those surveys. The arrangement of these trainings (including the vocational training) and the timing of data collection was such that their willingness to participate be highest: data collection was carried out during work hours; geographically away from their workplaces, co-workers and supervisors; there were no monetary obligations whatsoever to participate; voluntary participation and anonymity was granted, and there was no feedback of the results to their workplaces. 

### Questionnaire

The same self-report questionnaire was used during all 3 data collections that included demographic data, items on health status and mental health as follows. Scales and items of the questionnaire were taken from the Hungarian version of the European Health Interview Survey 2009 [21] or they were validated Hungarian versions of widely used scales [22]. 

#### Demographic data

These included year of birth from which age was calculated for the year of data collection; gender; education (in three categories: primary, secondary, tertiary); marital status (in two categories: lives alone/lives with partner); number of persons in the household; number of children; subjective wealth (five categories collapsed into two categories: bad (very bad and bad) or at least acceptable (acceptable, good, very good). Ethnic identity was self-reported on two items taken from the Hungarian census of 2011 [23]. Primary ethnicity was asked by the question of “Which ethnicity do you feel you belong to?”; secondary ethnicity by the question “Do you belong to another ethnicity?”. The answer listed all recognized minorities in Hungary including Roma from which respondents could choose. A dummy variable was created for Roma ethnic identity (yes/no). One question (yes/no) was related to completion of the vocational training. 

### Health status and health awareness

Self-rated health was measured on a 5-step Likert scale ranging from “very poor” to “excellent” and collapsed into 3 categories (very poor and poor as “poor”, acceptable as such, and good and excellent as “good”). Health problems limiting daily functioning in the past 6 months were assessed by a 3-step frequency scale (severely limiting, mildly limiting, no limitation) and collapsed into a binary variable (limitation vs no limitation). 

Health awareness was approximated by the question of “How much one can do for their health” that was answerable on a 4-step Likert scale from “nothing” to “very much”. Smoking was assessed by a 6-step frequency scale that was collapsed into two categories: nonsmoker and former smoker vs. current smoker. 

### Mental health

The short (12-item) version of GHQ had been used to detect psychological distress in the past weeks. Its use above the age of 17 years has been well established [24] and had been validated in Hungarian [22]. Answers are given on a Likert scale from 1 to 4 yielding a total score between 12 and 48. Another, simple scoring assigns a score of 1 to each present symptom, while lack of symptom is 0. A score above 4 identifies persons with high distress, a score of 4 or below average stress. This scoring yields a binary variable of 0 or 1, and had been used in Hungary to identify persons who are highly stressed [25]. 

Sense of coherence as a pervasive, enduring and dynamic feeling of confidence was defined by Antonovsky who also developed tools to measure the construct [26]. Of those, the short version validated in Hungarian [22] was used. Items can be answered on a 7-point Likert scale producing a total score between 13 and 91 where higher scores represent higher sense of coherence. 

The 17-item version [27] of the Dysfunctional Attitudes Scale (DAS) [28] was used to measure the presence and intensity of perfectionism and dependence as dysfunctional attitudes. DAS-A-17 is a self-report scale containing 17 items 11 of which is related to perfectionism, 6 items to dependence answerable on a 7-step Likert scale. The total score for perfectionism ranges between 6 and 42, for dependence between 11 and 77, higher scores representing more intense dysfunctional attitudes. 

Social support was measured by the Hungarian version of the Health and Lifestyle Survey and Health Survey for England [29]. Respondents answered seven questions on a 3-step frequency scale that yielded a total score between 7 and 21. Support from co-workers was estimated by one item answered on a 3-step scale (“My co-workers fully support me–not as much as I would like–not at all”). A binary variable for social support was created by dichotomizing responses to reflect optimal (full) support (score: 21) or less than optimal support (score <21). 

### Data analysis

Two-way tables of frequencies were used to analyse categorical variables across the study years, and the null hypothesis (no change across the years) was tested by Fisher’s exact test.

Normally distributed interval variables were described by the mean and standard deviation and were compared across the study years by analysis of variance. Non-normally distributed interval variables were described by the median and interquartile range and compared across the years by Kruskal–Wallis test. Correlation was tested by Spearman’s rank correlation test, Spearman’s rho and corresponding *p* values were calculated. The value of alpha was set at 0.05. Stata 16.1 and MS Excel 2013 were used for statistical analysis.

## Results

### Demographic data of health mediators

All positions for mediators were filled in October 2013, and 78% of the mediators self-identified as Roma. The number of health mediators varied during the Programme because some left and vacancies were filled. Altogether 58 different persons were employed during 5 years of the programme. The mean duration of employment was 32 months. However, only 4 persons joined the Programme more than 18 months after its start. Health mediators who completed vocational training in the Programme (*n* = 22) had been employed 7.2 months longer than those who did not (35.9 ± 8.4 vs 28.7 ± 14 months, *p*=0.017). 

The survey in 2013 had been restricted to those who were involved in vocational training in the Programme (*n* = 20, response rate: 90%). The survey was extended to all mediators in 2015 (*n* = 41, response rate = 100%) and 2017 (*n* = 32, response rate = 100%) who were employed at that time. Only two mediators entered the programme between 2015 and 2017 so with the exception of these 2 persons, all 32 respondents had also been among the respondents in 2015. Since the ethical permission of the survey did not allow for personal identification, it can only be stated that all respondents in 2017 were also employed in 2015. There was no significant difference in any demographic variables of the respondents in the 3 years of the surveys (Table 1).

**Table 1:** Demographic features of health mediators at the time of data collection

Variables	2013	2015	2017	*p*
Invited participants (*n*)	20	41	32	
Response rate (%)	90%	100%	100%	
Gender (female, %)	100%	95%	91%	0.590
Age (mean SD, years)	33.5 ± 7.6	37.3 ± 7.1	39.1 ± 8.4	0.061
Age range (min.–max. years)	18–44	22–57	19–59	–
Education				
Primary (%)	61	62	69	0.981
Secondary (%)	33	30	25	
Tertiary (%)	6	7	6	
Subjective wealth				
Bad (%)	44	39	41	0.946
Acceptable (%)	50	46	47	
Good (%)	6	15	12	
Lives with partner (%)	39	59	63	0.283
Number of children (median; IQR)	2 (1; 3)	2 (1; 2)	2 (1.5; 2.5)	0.982
Number of persons in household, median (IQR)	4 (3; 5)	4 (3; 4)	3 (3; 4)	0.116

Health mediators were dominantly young to middle-age women, approximately two-thirds of them with primary education, more than one-third in bad subjective wealth who raised on average 2 children, and lived in multiperson households (Table 1). The number of children varied between 0 and 5, and the number of persons living in the household ranged between at least 2 and no more than 7 

### Health status

Two-thirds of the respondents were in good or excellent health in 2013, and this did not significantly change by 2017. Similarly, there was no significant change in the proportion of those with mild or severe functional limitation.

Health awareness increased further, almost reaching significance (*p* = 0.079) though its degree had already been quite high even in 2013 (Table 2). Spearman correlation between health awareness, subjective health and functional limitation was carried out separately for 2015 and 2017. No significant correlation was found either between health awareness and subjective health (*p* = 0.059 in 2015, *p* = 0.310 for 2017) or health awareness and functional limitation (*p* = 0.698 for 2015, *p* = 0.059 for 2017).

Improving though nonsignificant trends could be observed in dysfunctional attitudes: the median for dependence decreased by 5 points, perfectionism 3.5 points. In terms of social support from family and friends, at least half of all respondents felt fully supported in each study year (Table 2). Support from co-workers was estimated by one item as described in “Methods”; more than three-fourths of mediators felt fully supported in each study year, and not a single person during 3 rounds of data collection responded that they did not feel supported at all by their co-workers.

**Table 2:** Health variables of health mediators

Variables	2013	2015	2017	*p*
Self-rated health (%)				
Good/excellent	67	61	65	0.537
Acceptable	20	31	32	
Bad/very bad	13	8	3	
Health problem causing at least mild functional limitation (%)	17	23	31	0.928
Health awareness (%)				
Can do much or very much for health	87	92	97	0.079
Can do little or nothing for health	13	10	6	
Sense of coherence (mean ± SD)	61.7 (± 14.6)	64.6 (± 11.8)	70.2 (± 12.8)	0.057
Highly stressed (%)	16.8	9.7	12.5	0.674
Dependence (median; IQR)	20 (11; 22)	15 (9; 21)	15 (9.5; 22.5)	0.513
Perfectionism (median; IQR)	23.5 (17; 38)	20 (17; 29.5)	20 (15; 25)	0.484
Social support, general				
Full (%)	50	60	73	0.333
Partial (%)	50	40	27	
Full social support from co-workers (%)	77.8	82.9	87.5	0.660
Smoking (current vs nonsmokers, %)	56 vs 44	54 vs 46	50 vs 50	0.688

### Smoking

44% of the mediators were daily smokers both in 2013 and, but this decreased considerably, though non-significantly, by 37% in 2017 (*p* = 0.688) (Table 2). Stratifying the group of nonsmokers to former smokers and never smokers, the prevalence of former smokers rose from 5.6% in 2013 to 12.5%. The proportion of never smokers was 38.9%, 36.6% and 37.5% in the subsequent study years (data not shown). 

### Mental health

Sense of coherence showed a mildly significant increase from 2013 to 2017 (*d* = 8.5 points, *p* = 0.057) (Table 2). However, when mediators were stratified by vocational training on-the-job, it was found that among those who did complete this training, a significant rise occurred in sense of coherence from 2015 to 2017 (*d* = 9.6, *p* = 0.033), but it remained unchanged among those who did not participate in this training (*d* = 0.8, *p* = 0.862) (Table 3).

**Table 3:** Indicators of mental health among health mediators by on-the-job vocational training

	2015	2017	*p* (2017 vs 2015)
Sense of coherence (mean ± SD)			
No vocational training	67.1 (± 11.9)	67.9 (13.5)	0.862
On-the-job vocational training	62.4 (± 12.1)	72 (± 10.8)	**0.033**
*p* (with vs without vocational training)	0.249	0.369	
High psychological distress (%)			
No vocational training	4.6	18.8	**0.016**
On-the-job vocational training	13.3	6.7	0.546
*p* (with vs without vocational training)	0.341	0.315	

Bold indicates *p* = significance level (P value). The significance level is the threshold for below which the null hypothesis is rejected even though by assumption it were true, and something else is going on

When psychological distress was analysed by completion of on-the-job vocational training, it was found that the proportion of highly stressed mediators did not significantly change (though it showed declining trend) in those who did (*d* = − 6.6%, *p* = 0.546), but significantly increased from 2015 to 2017 among those who did not participate in this training (*d* = 14.2%; *p* = 0.016) (Table 3). 

Sense of coherence of the mediators was also compared to sense of coherence of females obtained from population surveys representative of the Hungarian population [30] (since the overwhelming majority of mediators were females). Sense of coherence among health mediators in 2013 was significantly higher not only compared to females with primary education in the general population (*d* = 4.7; *p* < 0.001), but also compared to females in the general population (*d* = 1.4, *p* = 0.026) to whom they were most similar in terms of education. This difference became even greater by 2017 (difference compared to women with primary education: *d* = 11.7, *p* < 0.001; difference compared to females: *d *= 7.6, *p* < 0.001) (Table 4).

**Table 4:** Psychological distress and sense of coherence among health mediators compared to the general population

Variable	Group	2013	2017
High psychological distress (GHQ-12)	1. Health mediators (%)	16.7	12.5
	2. Females in the population (% ± 95% CI)	10.8 (8.2; 14.1)	6.8^a^ (4.7; 9.6)
	3. Females with primary education in the population (% ± 95% CI)	16.8 (11.8; 23.4)	13.7^a^ (8.07; 22.51)
*p *	1. vs 2	0.397	**< 0.001**
	1. vs 3	0.986	0.850
Sense of coherence (SoC-13)	4. Health mediators (mean ± SD)	61.8 (± 14.6)	70.2 (± 12.3)
	5. Females in the population (mean ± 95% CI)	60.4 (58.8; 61.9)	62.6^a^ (61.1; 64.2)
	6. Females with primary education in the population (mean ± 95% CI)	57.1 (55.1; 59.1)	58.5^a^ (55.3;61.7)
*p*	4. vs 5	**0.026**	**< 0.001**
	4. vs 6	**< 0.001**	**< 0.001**

Bold indicates *p* = significance level (P value). The significance level is the threshold for below which the null hypothesis is rejected even though by assumption it were true, and something else is going on

^a^Data are for 2019

The proportion of highly stressed health mediators decreased from 16.7% in 2013 to 12.5% in 2017 (Table 4), but this did not reach significance (*p* = 0.683). The proportion of highly distressed mediators was not significantly different from either compared to all females or females with primary education in the general population in 2013 (Table 4). However, the proportion of highly stressed mediators became significantly higher compared to all females (but was not different from their educational strata) in 2017 (Table 4). 

### Correlation between psychological stress (distress) and its potential determinants 

In order to identify determinants of psychological stress, correlation was examined among psychological distress and its potential determinants such as educational level, subjective wealth, sense of coherence, and social support as described in “Methods”. Data from 2015 and 2017 were pooled for this analysis. 

As shown in Table 5, sense of coherence proved to be the variable in the strongest negative correlation with distress, meaning that higher sense of coherence decreased the risk of being distressed. This variable showed significant positive relation with all other variables, allowing the conclusion that increased sense of coherence is related to higher level of education, greater subjective wealth, and having optimal social support. Greater subjective wealth and optimal social support are negatively related, that is, protective regarding distress according to our analysis.

**Table 5:** Correlation between some explanatory variables and psychological stress in the pooled data of 2015 and 2017

	Psychol. distress	Education	Subj. wealth	Sense of coh	Social supp
Psychological distress (yes/no)	1.000				
Education (primary, secondary, tertiary)	0.011 0.926	1.000			
Subjective wealth (poor, acceptable, good)	− 0.368 **0.002**	0.147 0.236	1.000		
Sense of coherence (33–89 points)	− 0.395 **0.001**	0.327 **0.007**	0.286 **0.019**	1.000	
Social support (optimal/less than optimal)	− 0.335 **0.005**	0.010 0.936	0.289 **0.018**	0.274 **0.025**	1.000

Spearman’s rho (Spearman correlation coefficients) and corresponding p values are shown in the table

Bold indicates *p* = significance level (P value). The significance level is the threshold for below which the null hypothesis is rejected even though by assumption it were true, and something else is going on

## Discussion

Our results showed improving trends in certain indicators of mental status of health mediators employed in the Primary Care Development Model Programme in Hungary between 2013 and 2017. Specifically, significant improvement in sense of coherence and distress was observed among those who obtained vocational qualification in the form of on-the-job training. The training focused on simple skills related to various diseases and pathological conditions (as needed in assistant nursing), but had also greatly increased the mediators’ awareness of their own health. By the end of the Model Programme, significant increase in sense of coherence was observed among those who completed vocational training as opposed to those who did not. The proportion of highly stressed mediators significantly increased among those who did not complete vocational training as opposed to those who did. Sense of coherence was found to be strongly and negatively related to being distressed, and it had a positive correlation with education, subjective wealth and social support. Since only two mediators entered the programme between 2015 and 2017 (who did not obtain vocational training because that finished by 2014), and since the number of those with vocational training obtained in the programme was identical in 2015 and 2017, we can state with certainty that improvement was not due to new mediators joining the programme who were in better health than those already in the programme.

Improving though nonsignificant changes in other health measures also reflected improvements such as decrease in dysfunctional attitudes, and decrease in smoking, in concert with a large body of research that has shown that employed individuals are healthier compared to those who are not [31, 32]. A notable finding was the very high level of social support among health mediators, especially the high level of feeling completely supported by their co-workers. Roma people, though far from heterogenous, tend to form closed groups especially in neighbourhoods where all members of the community know each other. Mediators in the same GP clusters lived in the area and most of them knew each other before joining the Programme. This likely contributed to the high level of support they experienced which was strengthened by working as a team within the healthcare team [33]. Social support played an important role in facilitating access to health care among Roma living in settlements in Slovakia [34]. Support from mediators can overcome lack of trust in healthcare and its providers among Roma people [35]. 

An advantage of our study is its follow-up design—the first such study on the health status of nonprofessional support workers employed in primary health care—in which all health mediators employed in the Model Programme were invited in 2015 and 2017. Another advantage of the study is their full participation in 2015 and 2017 that was probably due to the good working relations and mutual trust between mediators and the authors who had been responsible for the professional support of the mediators including all trainings.

One of the limitations of the study is the relatively low number of participants that resulted in the underpowering of statistical tests. This likely led to most results being underestimated, making significant results even more meaningful. Another shortcoming of the study is the partial involvement of health mediators in 2013 that was due to organizational and logistical limitations at the time of data collection. This prevented the creation of pooled data for all 3 years so that no regression analysis could be implemented.

The positive changes described in our paper provide evidence for the multiple benefits of on-the-job training for health mediators. Healthier workers provide better services and make better role models which is greatly needed in Roma communities [37, 38]. In addition to less favourable morbidity and mortality, Roma struggle with other issues, for example Roma patients suffering from coronary heart disease had higher levels of anxiety and lower sense of coherence compared to non-Roma patients [39]. 

The significantly increased distress of those who had no vocational training at the end of the Programme was probably due to the fact that there was uncertainty about their further employment, and those with vocational training had better job prospects. 

Education, employment, and health care constitute major areas for the development of human resources in all large-scale international frameworks aimed at disadvantaged populations. The Primary Care Model Programme of Hungary funded by the Swiss Contribution implemented activities in two of these areas (employment and health care) for the target population, and activities in all three areas for its Roma employees, the health mediators who became valued members of the primary care teams. Their contribution to the performance of the GP clusters such as the achievement of a very high participation rate in one of the new services (health status assessment) was detailed elsewhere [40]. 

The majority of mediators had been Roma women who tend to be in the most disadvantaged position in terms of education, employment, income and health status when compared to Roma men, or either gender groups of majority populations [42]. Positive changes in their health status, behaviour and health awareness, in addition to their meaningful work contribution [41] provides justification for the employment of health mediators in primary health care in those areas where large disadvantaged Roma population groups need to be cared for. 

## Conclusions

On-the-job vocational training improved the mental status of nonprofessional community workers (health mediators) in the long term. Specifically, the proportion of distressed persons decreased, sense of coherence increased among those who completed vocational programme at the beginning of their employment. The 5-year follow-up of mental health of mediators in the Primary Care Development Model Programme in Hungary revealed an improving trend in all investigated indicators between 2013 and 2017. A notable result was the significant increase in sense of coherence—a measure of mental resilience—among those mediators who completed vocational training as opposed to those who did not. In terms of high psychological stress, the opposite was found: the proportion of highly stressed mediators significantly increased among those who did not complete vocational training as opposed to those who did. Our results draw attention to the potential of improving mental health among health care workers who have been known to be under chronic job stress that may result in burnout, a workplace syndrome. This has been a growing public health concern among all types of health care workers affecting up to 35% of U.S. nurses and 54% of physicians. Burnout is a problem among all clinical disciplines and across care settings [43]. Since professional well-being is essential to providing high-quality patient care, any means are worthy of investigation by which the well-being of health care workers can be increased.

## Data Availability

The datasets generated and/or analysed during the current study are not publicly available due to containing personal and special data the availability and use of which is regulated by law (*2011. évi CXII. törvény az információs önrendelkezési jogról és az információszabadságról*). Data collected in the framework of the Programme are stored and managed by the National Public Health Institute of Hungary and available on reasonable request from the director of the Institute.
